# ASSOCIATION BETWEEN BODY WEIGHT PERCEPTION AND QUALITY OF DIET IN BRAZILIAN ADOLESCENTS

**DOI:** 10.1590/1984-0462/2020/38/2020057

**Published:** 2020-12-18

**Authors:** Mariana Migliavacca Madalosso, Beatriz Schaan, Felipe Vogt Cureau

**Affiliations:** a Graduate Program in Health Sciences: Cardiology and Cardiovascular Sciences, Universidade Federal do Rio Grande do Sul, Porto Alegre, RS, Brazil.

**Keywords:** Adolescent, Body image, Body weight, Food consumption, Diet, Adolescente, Imagem corporal, Peso corporal, Consumo de alimentos, Dieta

## Abstract

**Objective:**

To evaluate the association between body weight perception and quality of diet among Brazilian adolescents.

**Methods:**

The sample was composed of 71,740 adolescents aged from 12 to 17 years-old enrolled in the Study of Cardiovascular Risks in Adolescents (*Estudo de Riscos Cardiovasculares em Adolescentes* - ERICA), carried out during 2013-2014. Body weight perception was self-reported. Food consumption was assessed by food record and quality of diet index for Brazilian adolescents (DQIA-BR) was calculated, considering the balance, diversity, and diet composition. The quality of diet was compared according to weight perception for the entire sample and after stratification by nutritional status. Linear regression models were used to assess the association between body weight perception and quality of diet.

**Results:**

Among the studied adolescents, 14.7 and 30.3% reported to be underweight or overweight in relation to their desired weight, respectively. Those who perceived themselves as overweight had lower quality of diet (DQIA-BR=16.0 vs. 17.4 points; p<0.001). After stratification by BMI, adolescents with normal weight (DQIA-BR=15.3 points) or overweight (DQIA-BR=16.1 points), but who perceived themselves as overweight showed lower quality of diet when compared to their peers. In adjusted analysis, overweight perception (β= -0.51; 95%CI -0.77; -0.24) was associated to lower quality of diet. However, this association was no longer significant after stratification by BMI status.

**Conclusions:**

Body weight perception can influence the consumption of healthy foods and the quality of diet, especially for those who consider themselves overweight. However, this association is influenced by nutritional status.

## RESUMO

**Objetivo:**

Avaliar a associação entre percepção de peso corporal e qualidade da dieta de adolescentes brasileiros.

**Métodos:**

A amostra estudada foi composta de 71.740 adolescentes de 12-17 anos participantes do Estudo de Riscos Cardiovasculares em Adolescentes (ERICA) realizado entre 2013 e 2014. A percepção do peso corporal foi avaliada por questionário autoaplicável; o consumo alimentar, por recordatório alimentar; e a qualidade da dieta pelo índice de qualidade da dieta de adolescentes brasileiros (IQDA-BR), considerando o equilíbrio, a diversidade e a composição da dieta. A qualidade da dieta foi comparada de acordo com a percepção do peso para toda a amostra e após estratificação por índice de massa corpórea (IMC). Modelos de regressão linear foram utilizados para avaliar a associação entre distúrbios na percepção do peso e qualidade da dieta.

**Resultados:**

Entre os adolescentes, 14,7 e 30,3% percebiam-se abaixo ou acima do peso, respectivamente. Aqueles que se percebiam acima do peso apresentaram menor qualidade da dieta (IQDA-BR=16,0 *vs*. 17,4 pontos; p<0,001). Após estratificação por IMC, adolescentes com peso normal (IQDA-BR=15,3 pontos) ou excesso de peso (IQDA-BR=16,1 pontos), mas que se percebiam como acima do peso, apresentaram pior qualidade da dieta se comparados ao demais. Em análise ajustada, perceber-se acima do peso (β= -0,51; intervalo de confiança de 95% - IC95% -0,77; -0,24) foi associado a pior qualidade da dieta, porém essa associação perdeu significância após a estratificação por IMC.

**Conclusões:**

A percepção do peso corporal parece influenciar o consumo de alimentos saudáveis e a qualidade da dieta principalmente daqueles que se consideram acima do peso, contudo essa associação é influenciada pelo estado nutricional.

## INTRODUCTION

Over the last 50 years, Brazilian population’s nutritional profile has been drastically modified, which has been characterized by a reduction in the prevalence of malnutrition and a progressive increase in the prevalence of overweight and obesity, even among children and adolescents.^1^ Results of the Study of Cardiovascular Risks in Adolescents (*Estudo de Riscos Cardiovasculares em Adolescentes* - ERICA) carried out between 2013 and 2014 point to the prevalence of 17.1 and 8.4% of overweight and obesity, respectively, among Brazilian adolescents aged 12-17.^2^ Worsening of quality of diet and increase in the amount of calories consumed in the Brazil has been observed since the 1970s, and, among adolescents, there is a high consumption of sweetened beverage and foods of low nutritional value.^3^ ^,^ ^4^

Among adolescents, concerns about body image potentially related to changes in body mass index (BMI) are increasingly frequent.^5^ Body image is a multifaceted construct that involves a person’s perceptions, thoughts, feelings, and behaviors in relation to the size, shape, and structure of their body, and there may be distortions between body weight perception and objective measures.^6^ In Brazil, results from the National School Health Survey (*Pesquisa Nacional de Saúde do Escolar* - PeNSE) show that about 40% of adolescents have disorders of body image,^7^ which play an important role in the development of obesity^8^ and eating disorders.^9^

During adolescence, food preferences are defined and, because of the progressive gain in autonomy, exposing individuals to foods of poorer nutritional quality.^10^ Previous studies highlight the possible relation of disorders in the body weight perception with the adoption of diet behaviors and unhealthy eating habits among adolescents.^5^ ^,^ ^11^ ^,^ ^12^

In this context, an alternative that is gaining attention is the evaluation of diet composition with quality of diet indexes, a method that can quantitatively identify changes in the quality of diet. The use of these indexes follows the epidemiological tendency of assessing dietary patterns instead of nutrients in isolation, because it can better represent the diet and its impact on the health of populations.^13^ ^,^ ^14^ Recently, Ronca et al.^15^ proposed the quality of diet index of Brazilian adolescents (DQIA-BR), an adaptation of an European index for the Brazilian food culture.

The association between disorders in the body weight perception and dietary quality indicators among adolescents is a topic little explored. The hypothesis of the present study was that dissatisfied adolescents or those with inadequate weight perception have a worse quality of diet when compared to their peers who do not have such disorders. Therefore, the objective of present study was to evaluate the association between body weight perception and the quality of diet of Brazilian adolescents.

## METHOD

ERICA is a multicenter, national, and school-based cross-sectional study. Its target population was stratified in 32 geographic strata of the country, of them 27 capitals and five other strata with other municipalities with over than 100 thousand inhabitants from each of Brazil’s macro-regions (Northeast, North, Midwest, Southeast, and South). In each stratum, schools were selected with a probability proportional to the number of students enrolled between the 7^th^ year of elementary school and the 3^rd^ year of high school and inversely the distance between the school and the state capital. In each school, three combinations of year and shift (morning or afternoon) were selected. All students in each of the selected classes were invited to participate in the study.

The sample consisted of adolescents aged 12 to 17 years old, enrolled in public and private schools, in 273 eligible Brazilian municipalities. Data collection took place in schools between 2013 and 2014 by trained professionals. The study questionnaire was answered by adolescents in an electronic data collector (personal digital assistant - PDA) in the presence of a researcher. In addition to the questionnaire, they answered a 24-hour food recall (24hR) applied in the form of an interview with the help of a software developed for the study,^16^ as well as undergoing anthropometric assessment in a private space provided by the school. Details of sampling and data collection were previously published.^17^ ^,^ ^18^

Adolescents’ food intake estimate was calculated using an 24hR, which was applied to the entire sample. Subsequently, a second 24hR was applied to a subsample selected at random from about 10% of the total sample, which allowed considering intrapersonal variability when estimating the usual food consumption of adolescents.^19^ ^,^ ^20^

To analyze the quality of adolescents’ diet, the DQIA-BR was used, an instrument adapted for the Brazilian population based on the European adolescents’ quality of diet quality (DQI-A).^15^ ^,^ ^21^ The DQIA-BR analyzes balance, diversity, and composition of diet, which are the three principles of healthy eating,^15^ and does not consider the degree of food processing in its classification.

DQIA-BR assesses 10 food groups, categorized into recommended food groups and non-recommended food groups. The recommended food groups are:

- Breads, potatoes, and grains.
- Vegetables.
- Fruits.
- Dairy products.
- Cheeses.
- Meat, fish, and eggs.
- Legumes.
- Oils and fats.

The food groups which are not recommended are:

- Snacks and sweets.
- Sweetened beverage, fruit juices, and alcoholic drinks.

For each food group, the recommendation of specific daily intake for adolescents was followed.^22^ ^,^ ^23^ The food groups were analyzed based on the three main components (quality, diversity, and balance), which had different weights; the quality of diet was twice the weight of the other components. The total score of DQIA-BR ranges from -33 to 100 points; in this index, the highest score represents better quality of diet. More information on the construction of DQIA-BR can be found in another publication.^15^

Anthropometric measurements were performed with participants wearing light clothing and without shoes. Height was measured with a portable stadiometer (Alturexata^®^, Minas Gerais, Brazil), and body weight was measured with a digital scale (model P150m, 200 kg capacity, Líder^®^, São Paulo, Brazil). BMI was calculated by dividing weight (kg) by the square of height (m^2^). The World Health Organization reference curves for BMI specific for age and sex were used to classify the nutritional status of adolescents.^24^ For analysis purposes, the categories were grouped into:

- Low weight: Z score of BMI<-1.
- Normal weight: Z score of BMI≥-1 and ≤1.
- Overweight/obesity: Z score of BMI>1.

Weight perception was assessed with the question: Are you satisfied with your weight? The answer options were *yes* and *no*. Adolescents who answered *yes* to this question were considered satisfied with their own weight. For those who answered *no*, the following question was used: In your opinion, what is your weight like? The response options were *under the ideal*, *over the ideal*, or *far over the ideal*. Thus, adolescents were classified into three categories, according to their body weight perception:

- Satisfied.
- Under the ideal weight.
- Over the ideal weight.

Other variables used in this study were: sex, age (in complete years), self-reported skin color (white, black, brown, and others), geographic region (North, Northeast, Southeast, South, and Midwest) and maternal education. The socioeconomic level was assessed using the Brazil criterion of the Brazilian Association of Research Companies (*Associação Brasileira de Empresas de Pesquisa*), which considers the amount of goods at home, the presence of a domestic worker, and the education of the head of the family.^25^ The score obtained can vary from 0 to 46 points, with the highest number of points indicating better economic conditions. For analysis purposes, this score was divided into tertiles.

All variables studied were described using mean or proportion, with their respective 95% confidence intervals (95% CI). Heterogeneity in food consumption according to weight perception and nutritional status was assessed with the F (ANOVA) statistic, and the difference between the categories with the non-overlapping of the 95%CI - depending on sample size, this can be considered a conservative alternative to traditional statistical tests.

Multiple linear regression models were used to assess the association between the categories of perceived body weight, and the overall quality of diet and its components. Estimates of regression coefficients and their respective 95%CI were presented in a model adjusted for sex, age, skin color, economic condition, region, and maternal education. Analyzes were performed for the total sample and stratified by the nutritional status of the adolescents, given that this may be associated to variations in the perception of weight and food.^5^ The reference category was the one that represents adequacy between the measured and perceived body weight (for example, normal weight adolescents satisfied with their weight), thus estimating β value for those who had some degree of distortion between weight perception and BMI classification.

All analyzes were performed with Stata version 14.0. The set of *svy* commands was used to consider the complex sample design and represent the set of the studied adolescent population. All tests were two-tailed, and significance level of 5% was adopted.

The present study was conducted in accordance with the principles of the Declaration of Helsinki. All adolescents agreed in writing to participate in the study (consent form); in five states, a free and informed consent form signed by the parent or legal guardian was also requested, as determined by the respective Research Ethics Committee (CEP) or the state Department of Education to participate in the study. ERICA was approved at the CEPs of all participating centers.

## RESULTS

In all, 71,740 adolescents made up the studied sample. Table 1 presents the characteristics of the sample and the distribution of adolescents’ weight perception according to the covariates. Most sample consisted of female adolescents, between 15 and 17 years old, of brown skin color, and living in the Northeastern or Southeastern regions of the country. One in four adolescents was classified with overweight/obesity. Regarding body weight perception, 55% (95%CI 53.9-56.0) of adolescents said they were satisfied with their weight. Among those dissatisfied with weight, 14.7% (95%CI 14.3-15.2) perceived themselves as underweight, whereas 30.3% (95%CI 29.2-31.5) reported feeling overweight.

**Table 1:** Sample characteristics according to the perception of body weight, Study of Cardiovascular Risks in Adolescents 2013-2014.

	Body weight perception			
	Total sample	Satisfied	Dissatisfied	
			Underweight	Overweight
	n=71,740	n=37,226	n=12,194	n=22,320
Sex				
Female	55.5 (55.1-55.8)	46.2 (44.7-47.8)	14.5 (13.8-15.2)	39.3 (37.6-41.0)
Male	44.5 (44.2-44.9)	63.6 (62.3-64.9)	14.9 (14.3-15.6)	21.4 (20.3-22.7)
Age (years old)				
12-14	45.9 (45.5-46.3)	57.3 (55.7-59.0)	12.5 (11.9-13.1)	30.2 (28.6-31.9)
15-17	54.1 (53.7-54.5)	52.3 (51.1-53.6)	17.2 (16.6-17.9)	30.4 (29.1-31.8)
Skin color				
White	39.0 (37.4-40.7)	53.6 (51.9-55.2)	13.8 (13.1-14.6)	32.6 (30.9-34.3)
Black	8.1 (7.4-8.8)	58.4 (55.4-61.4)	13.6 (12.0-15.3)	28.0 (25.0-31.2)
Brown	47.5 (46.2-48.9)	55.3 (54.0-56.5)	15.5 (14.7-16.3)	29.2 (27.9-30.5)
Other (Indigenous)	5.4 (5.0-5.8)	57.2 (53.7-60.6)	15.8 (13.7-18.2)	27.0 (23.4-30.9)
Region in Brazil				
Northern	20.3 (19.9-20.5)	55.0 (53.7-56.3)	19.8 (18.8-20.9)	25.2 (24.0-26.3)
Northeastern	31.0 (30.7-31.4)	53.4 (51.6-55.3)	18.7 (17.6-19.8)	27.9 (25.8-30.1)
Southeastern	23.0 (22.7-23.3)	56.4 (54.4-58.3)	12.8 (12.1-13.5)	30.9 (28.9-32.9)
Southern	12.7 (12.5-13.0)	52.7 (51.1-54.2)	12.1 (11.1-13.1)	35.2 (33.7-36.8)
Central-Western	13.0 (12.8-13.3)	53.5 (52.1-54.9)	15.0 (14.1-15.9)	31.5 (30.2-32.8)
Socioeconomic level (score)				
Tertile 1 (5-21)	32.1 (30.7-33.5)	55.0 (53.5-56.5)	17.0 (15.9-18.1)	28.0 (26.5-29.5)
Tertile 2 (22-27)	37.2 (36.3-38.1)	55.0 (53.5-56.5)	15.0 (13.9-16.1)	30.0 (28.4-31.7)
Tertile 3 (28-46)	30.7 (29.1-32.4)	55.1 (52.9-57.3)	12.1 (11.1-13.1)	32.8 (31.0-34.7)
Maternal education				
Incomplete elementary school	19.4 (17.5-21.4)	56.2 (54.3-58.0)	16.3 (15.2-17.4)	27.6 (25.6-29.6)
Complete elementary school/ incomplete high school	13.6 (12.8-14.4)	54.8 (52.8-56.8)	15.3 (14.1-16.6)	29.9 (28.1-31.8)
Complete high school	17.4 (16.2-18.8)	51.2 (49.3-53.1)	14.7 (13.6-15.8)	34.1 (32.3-36.0)
Incomplete and complete university	20.2 (18.1-22.5)	53.2 (51.5-55.0)	14.5 (13.5-15.6)	32.3 (30.7-34.0)
Did not inform	29.4 (28.0-30.9)	58.1 (55.9-60.2)	13.6 (12.6-14.6)	28.4 (26.6-30.2)
Nutritional status				
Underweight	2.8 (2.6-3.1)	43.6 (39.3-48.1)	54.5 (50.0-58.9)	1.9 (1.2-2.9)
Normal weight	71.8 (70.6-72.9)	65.9 (64.8-66.9)	17.1 (16.6-17.7)	17.0 (16.0-18.0)
Overweight/obesity	25.4 (24.3-26.6)	25.4 (24.1-26.8)	3.5 (3.0-4.1)	71.1 (69.7-72.4)

Results are expressed in % (95% CI). ERICA: Study of Cardiovascular Risks in Adolescents (*Estudo de Riscos Cardiovasculares em Adolescente*); 95%CI: 95% confidence interval; underweight: Z score of BMI<-1; normal weight: Z score of BMI≥ -1 and ≤1; overweight/obesity: Z score of BMI >1.

Table 2 shows the distribution of the quality of diet and of the food groups evaluated according to the perception of the adolescents’ body weight. The quality of diet for all groups was low, but it was even lower among adolescents who considered themselves overweight. Among adolescents with perceived weight above the ideal, there was a lower consumption of food from the groups of breads, potatoes, cereals, dairy products, meat, fish, and eggs, oils and fats, legumes and snacks, and sweets; and a higher consumption of vegetables and cheeses compared to the others. Adolescents who believed they were underweight consumed more bread, potatoes, cereals, snacks, and sweets compared to those with a different weight perception.

**Table 2:** Mean consumption of food groups and quality of diet, according to the perception of body weight by Brazilian adolescents, Study of Cardiovascular Risks in Adolescents 2013-2014.

Food consumption (g)	Body weight perception			p-value*
	Satisfied	Dissatisfied		
		Underweight	Overweight	
Breads, potato, and cereals	384.5 (382.6-386.4)	398.4 (395.6-401.1)	339.9 (336.8-343.1)	<0.001^a.b.c^
Vegetables	52.2 (51.5-53.0)	51.8 (51.1-52.5)	56.0 (55.1-56.9)	<0.001^b.c^
Fruits	42.3 (40.9-43.7)	43.1 (41.9-44.3)	44.4 (43.2-45.6)	0.164
Dairy products	184.6 (182.9-186.2)	186.6 (184.5-188.7)	169.7 (167.8-171.7)	<0.001^b.c^
Cheeses	7.9 (7.7-8.0)	8.1 (7.9-8.4)	8.3 (8.1-8.6)	0.001^b^
Meat, fish, and eggs	191.6 (190.9-192.2)	192.8 (191.8-193.8)	180.5 (179.5-181.4)	<0.001^b.c^
Oils and fats	5.3 (5.2-5.3)	5.4 (5.3-5.5)	4.4 (4.3-4.5)	<0.001^b.c^
Legumes	183.4 (180.6-186.1)	182.9 (180.2-185.5)	151.8 (148.4-155.1)	<0.001^b.c^
Snacks and sweets	92.9 (91.9-93.9)	102.6 (101.4-103.7)	83.2 (82.4-84.0)	<0.001^a.b.c^
Sweetened beverage	515.5 (507.8-523.2)	514.5 (504.4-524.6)	507.6 (501.4-513.7)	0.161
DQIA-BR (points)	17.4 (17.2-17.6)	17.4 (17.1-17.7)	16.0 (15.7-16.2)	<0.001^b.c^

Results are expressed in Mean (95%CI). ERICA: Study of Cardiovascular Risks in Adolescents (*Estudo de Riscos Cardiovasculares em Adolescentes*); DQIA-BR: quality of diet index of Brazilian adolescents; *ANOVA test for heterogeneity; differences between categories were determined by the non-overlapping of the 95% confidence intervals (95% CI): ^a^satisfied *versus* underweight; ^b^satisfied *versus* overweight; ^c^underweight *versus* overweight.

Average consumption in the evaluated food groups and the quality of diet according to body weight perception and nutritional status are shown in Table 3. There was no difference in food consumption or in the quality of diet of underweight adolescents, regardless of weight perception. Among eutrophic adolescents, those who considered themselves overweight (15.7 points; 95%CI 15.3-16.1) had worse quality of diet compared to the others. Among overweight adolescents, those who perceived themselves as overweight showed lower consumption of most food groups recommended for healthy eating and, consequently, poorer quality of diet (16.1 points; 95%CI 15.9-16.4), but only if compared to those satisfied with their own weight.

**Table 3:** Mean consumption of food groups and quality of diet according to the perception of body weight and nutritional status of Brazilian adolescents, Study of Cardiovascular Risks in Adolescents 2013-2014.

Food consumption (g)	Body weight perception			
	Satisfied	Dissatisfied		p-value*
		Underweight	Overweight	
Underweight				
Breads, potato, and cereals	433.2 (425.6-440.7)	423.9 (416.4-431.4)	431.2 (408.0-454.4)	0.266
Vegetables	46.4 (44.6-48.2)	49.0 (47.1-50.9)	53.2 (44.8-61.6)	0.114
Fruits	39.8 (36.8-42.9)	38.0 (35.9-40.1)	39.5 (32.0-47.0)	0.586
Dairy products	206.0 (199.9-212.1)	201.9 (196.2-207.7)	196.9 (187.1-206.6)	0.280
Cheeses	7.2 (6.8-7.7)	7.5 (7.1-7.9)	7.1 (6.1-8.2)	0.457
Meat, fish, and eggs	196.8 (193.6-200.0)	194.1 (191.2-197.1)	195.3 (185.4-205.2)	0.552
Oils and fats	6.5 (6.2-6.8)	6.1(5.9-6.3)	6.4 (5.8-7.0)	0.072
Legumes	212.7 (205.1-220.3)	200.2 (193.5-206.9)	218.2 (198.3-238.1)	0.052
Snacks and sweets	109.9 (106.5-113.4)	115.7 (111.8-119.6)	109.9 (98.3-121.5)	0.088
Sweetened beverage	483.8 (460.6-507.0)	478.5 (448.2-508.7)	406.1(297.9-514.2)	0.389
DQIA-BR (points)	19.1 (18.4-19.7)	18.8 (18.0-19.6)	21.1 (17.4-24.8)	0.474
Normal weight				
Breads, potato, and cereals	386.1 (384.1-388.2)	399.4 (396.6-402.2)	345.0 (339.8-350.2)	<0.001^a.b^
Vegetables	52.1 (51.4-52.9)	52.3 (51.5-53.1)	54.5 (53.5-55.5)	<0.001^b.c^
Fruits	42.3 (40.8-43.8)	43.7 (42.4-45.0)	44.2 (42.3-46.1)	0.113
Dairy products	185.3 (183.6-187.1)	185.8 (183.5-188.0)	175.3(172.8-177.8)	<0.001^b.c^
Cheeses	7.8 (7.7-7.9)	8.2 (8.0-8.4)	8.0 (7.6-8.3)	0.007^a^
Meat, fish, and eggs	191.2 (190.4-192.0)	193.0 (191.8-194.2)	176.7 (175.2-178.2)	<0.001^b.c^
Oils and fats	5.3 (5.2-5.4)	5.4 (5.3-5.4)	4.5 (4.4-4.7)	<0.001^b.c^
Legumes	184.1 (181.2-186.9)	182.3 (179.4-185.1)	153.2(149.0-157.3)	<0.001^b.c^
Snacks and sweets	94.5 (93.3-95.7)	102.8 (101.6-104.0)	92.2 (90.9-93.6)	<0.001^a^
Sweetened beverage	515.1 (506.8-523.4)	519.5 (509.3-529.6)	493.3(482.6-504.0)	0.005^b.c^
DQIA-BR (points)	17.4 (17.1-17.6)	17.3 (17.0-17.6)	15.7 (15.3-16.1)	<0.001^b.c^
Overweight/obesity				
Breads, potato, and cerelas	363.7 (360.5-366.9)	340.3 (328.6-352.0)	336.3 (333.3-339.2)	<0.001^a.b^
Vegetables	53.9 (52.4-55.5)	50.4 (48.0-52.9)	57.0 (55.9-58.1)	<0.001^b.c^
Fruits	42.5 (40.6-44.4)	43.5 (39.5-47.5)	44.5 (43.2-45.8)	0.190
Dairy products	175.0 (171.6-178.5)	171.3 (162.0-180.6)	165.9 (163.8-168.0)	<0.001^b^
Cheeses	8.5 (8.1-8.8)	8.5 (7.2-9.8)	8.5 (8.2-8.8)	0.977
Meat, fish, and eggs	193.1 (191.6-194.7)	187.7 (182.5-192.8)	183.0 (181.7-184.2)	<0.001^b^
Oils and fats	4.8 (4.7-4.9)	4.4 (4.1-4.6)	4.3 (4.2-4.4)	<0.001^a.b^
Legumes	172.9 (168.9-176.9)	162.0 (155.0-169.1)	150.6 (147.0-154.3)	<0.001^b.c^
Snacks and sweets	78.2 (76.9-79.4)	76.0 (72.7-79.3)	77.0 (76.0-78.0)	0.295
Sweetened beverage	524.1 (509.9-538.2)	508.1 (465.2-551.0)	517.4 (508.0-526.9)	0.606
DQIA-BR (points)	17.4 (17.0-17.8)	16.5 (15.3-17.7)	16.1 (15.9-16.4)	<0.001^b^

Results are expressed in Mean (95%CI). ERICA: Study of Cardiovascular Risks in Adolescents (*Estudo de Riscos Cardiovasculares em Adolescentes*); DQIA-BR: quality of diet index of Brazilian adolescents; *ANOVA test for heterogeneity; differences between categories were determined by the non-overlapping of the 95% confidence intervals (95%CI): ^a^satisfied *versus* underweight; ^b^satisfied *versus* overweight; ^c^underweight *versus* overweight.

Not being satisfied for perceiving oneself overweight (β=-0.51; 95%CI -0.77; -0.24) was associated to the poorest quality of diet in the sample (Table 4). After stratification by nutritional status, there was no association between quality of diet and differences in body weight perception (Table 4). However, lower consumption of foods recommended as healthy was associated to the perception of being overweight among eutrophic and being satisfied with their weight for those overweight/obese.

**Table 4:** Multiple linear regression models for the association between body weight perception and quality of diet in the total sample and stratified by nutritional status, Study of Cardiovascular Risks in Adolescents 2013-2014.

		Body weight perception		
		Satisfied	Dissatisfied	
			Underweight	Overweight
Total sample	Food consumption (g)			
	Breads, potato, and cereals	Ref.	9.18 (6.92-11.44)***	-13.25 (-15.67; -10.82)***
	Vegetables	Ref.	-0.45 (-1.16-0.27)	0.85 (2.00-0.04)*
	Fruits	Ref.	0.01 (-1.39-1.41)	1.11 (-0.30-2.52)
	Dairy products	Ref.	5.73 (3.94-7.52)***	-4.43 (-6.20; -2.67)***
	Cheeses	Ref.	0.06 (-0.11-0.22)	0.39 (0.20-0.57)***
	Meat, fish, and eggs	Ref.	1.05 (0.33-1.77)**	-0.69 (-1.19; -0.20)**
	Oils and fats	Ref.	0.12 (0.06-0.18)***	-0.22 (-0.27; -0.17)***
	Legumes	Ref.	3.02 (1.74-4.30)***	-7.56 (-8.96; -6.15)***
	Snacks and sweets	Ref.	5.21 (4.08-6.34)***	-4.08 (-4.95; -3.22)***
	Sweetened beverage	Ref.	-1.94 (-11.06-7.18)	-8.09 (-18.84-2.66)
	DQIA-BR (points)	Ref.	0.25 (-0.04-0.55)	-0.51 (-0.77; -0.24)***
Underweight	Breads, potato, and cereals	1.27 (-5.36-7.90)	Ref.	7.96 (-11.88-27.79)
	Vegetables	-0.83 (-3.19-1.54)	Ref.	2.61 (-3.38-8.59)
	Fruits	2.79 (-1.02-6.60)	Ref.	-1.64 (-6.56-3.28)
	Dairy products	-3.69 (-11.31-3.93)	Ref.	-4.16 (-19.07-10.75)
	Cheeses	-0.54 (-0.89; -0.20)**	Ref.	0.11 (-0.98-1.21)
	Meat, fish, and eggs	-1.63 (-3.90-0.64)	Ref.	1.63 (-3.96-7.22)
	Oils and fats	0.11(-0.11-0.33)	Ref.	-0.01 (-0.47-0.45)
	Legumes	1.74 (-2.43-5.91)	Ref.	6.18 (-2.23-14.59)
	Snacks and sweets	-3.16 (-7.74-1.42)	Ref.	-0.09 (-11.71-11.53)
	Sweetened beverage	1.37 (-29.76-32.51)	Ref.	-4.38 (-85.86-77.10)
	DQIA-BR (points)	-0.23 (-1.16-0.70)	Ref.	-0.15 (-2.21-1.91)
Normal weight	Breads, potato, and cereals	Ref.	10.84 (8.55-13.13)***	-15.70 (-19.20; -12.19)***
	Vegetables	Ref.	-0.41 (-1.22-0.40)	0.39 (-0.48-1.26)
	Fruits	Ref.	0.22 (-1.29-1.73)	0.81 (-1.18-2.80)
	Dairy products	Ref.	6.26 (4.24-8.29)***	-5.45 (-7.42; -3.48)***
	Cheeses	Ref.	-0.01 (-0.19-0.16)	0.48 (0.23-0.73)***
	Meat, fish, and eggs	Ref.	1.09 (0.26-1.92)**	-1.04 (-1.66; -0.42)***
	Oils and fats	Ref.	0.14 (0.07-0.21)***	-0.25 (-0.32; -0.18)***
	Legumes	Ref.	3.66 (2.28-5.03)***	-8.39 (-10.36; -6.42)***
	Snacks and sweets	Ref.	5.68 (4.51-6.85)***	-4.42 (-5.54; -3.31)***
	Sweetened beverage	Ref.	-0.24 (-8.67-8.20)	-11.96 (-26.72-2.79)
	DQIA-BR (points)	Ref.	0.15 (-0.13-0.42)	-0.27 (-0.71-0.17)
Overweight/obesity	Breads, potato, and cereals	11.77 (9.20-14.35)***	0.89 (-9.26-11.04)	Ref.
	Vegetables	-1.58 (-3.16; -0.01)*	-4.89 (-8.02; -1.77)	Ref.
	Fruits	-2.44 (-4.96-0.07)	-2.02 (-6.28-2.23)	Ref.
	Dairy products	3.87 (-0.19-7.93)	2.64 (-3.32-8.60)	Ref.
	Cheeses	-0.39 (-0.74; -0.04)	0.52 (-0.45-1.49)	Ref.
	Meat, fish, and eggs	0.1(-0.70-1.02)	0.24 (-1.68-2.15)	Ref.
	Oils and fats	0.26 (0.18-0.34)***	0.03 (-0.16-0.23)	Ref.
	Legumes	7.90 (5.84-9.95)***	2.78 (-1.33-6.88)	Ref.
	Snacks and sweets	3.99 (2.32-5.66)***	2.49 (-0.59-5.56)	Ref.
	Sweetened beverage	-3.32 (-18.66-12.02)	-4.91 (-45.84-36.01)	Ref.
	DQIA-BR (points)	0.28 (-0.22-0.78)	-0.14 (-1.52-1.23)	Ref.

Results are expressed in adjusted β (95%CI). ERICA: Study of Cardiovascular Risks in Adolescents (*Estudo de Riscos Cardiovasculares em Adolescentes*); DQIA-BR: quality of diet index of Brazilian adolescents; Ref: reference groups represent adolescents who reported adequate perception of weight in relation to the nutritional status assessed with body mass index; Wald test for heterogeneity in relation to the reference category: *p <0.05; **p <0.01; ***p <0.001.

## DISCUSSION

The present study evaluated the influence of body weight perception on the quality of adolescents’ diet. The quality of diet in the sample was low, even lower among those who perceived themselves overweight. The greatest variations in the consumption of the evaluated food groups were observed in eutrophic adolescents, but who considered themselves underweight or overweight, followed by those with overweight/obesity, but who reported being satisfied with their weight.

Adolescents who considered themselves overweight had lower consumption of recommended foods, which resulted in poorer quality of diet, even though they showed lower consumption of sweets and snacks (non-recommended foods). On the other hand, adolescents who perceived themselves underweight had a higher consumption of food in the groups of breads, potatoes, and cereals, sweets and snacks. A previous study showed that behaviors such as eating less, cutting off calories, or avoiding fatty foods are strategies to maintain or lose weight.^5^ In contrast, adolescents who considered themselves underweight were less concerned with reaching the ideal weight compared to those who perceived themselves overweight.^5^ It is believed that, when perceiving themselves as thin, adolescents understand themselves as healthy and neglect their food choices, in addition to not adopting other healthy behaviors, which can cause cardiometabolic changes regardless of BMI.^26^

After stratifying the sample according to the BMI categories, lower DQIA-BR values were found in adolescents with normal weight who overestimated their weight (perceived themselves as overweight) and among those with overweight/obesity with correct perception of their weight (they perceived themselves overweight), when compared to their peers satisfied with their weight. These results emphasize that eutrophic or overweight/obese adolescents who perceive themselves as overweight should receive special attention in relation to food, because only reducing consumption, often of healthy foods, should not be the priority alternative.^27^

In the present study, after adjusting for possible confounding factors, a worse quality of diet was observed among adolescents who perceived themselves as overweight. In additional analyzes, after stratification by BMI category, this association lost significance, which suggests an important role for nutritional status in the relation between weight perception and quality of food. In addition, in the stratified analysis, a higher consumption of food groups considered to be more palatable was observed (for example, snacks and sweets; breads, potatoes, and cereals) among adolescents with underestimated body weight perception, which may suggest the adoption of a strategy for weight gain or inadequate assessment of food choice associated with weight perception.^28^ ^,^ ^29^

Studies involving overweight adolescents show that the greater the degree of dissatisfaction with weight, the greater the chances that these individuals will follow a more restrictive eating pattern, characterized by greater consumption of whole grains, vegetables, and fruits.^29^ These adolescents also are less likely to follow a Western dietary pattern, consisting of sweets, soft drinks, fast food, beef, and dairy products.^29^ In another study, the desire to be thinner was associated with the restriction of some foods, as well as the total caloric value of the diet.^28^ Our results reinforce the importance of perceiving weight in food choices, variable that should be considered in dietary interventions involving adolescents.

The results of the present study may have clinical and public health implications. Weight perception seems to have an important role in the adolescents’ food choices, but more attention should be given to adolescents who perceive themselves overweight, given that they present poorer quality of diet and tend to reduce the consumption of food groups recommended as healthy. These results may indicate the practice of self-modification of diet. That is, weight perception can be an indicator of changes in eating behaviors. In addition, our results indicate that the assessment of weight perception should not be disassociated from the nutritional status of adolescents. The combination of such information can assist in conducting effective interventions aimed at improving the quality of diets among Brazilian adolescents, which is considered low overall.^15^

The study has some limitations. Because it is a cross-sectional study, we cannot rule out the possibility of reverse causality. ERICA is a school-based study, so we cannot extrapolate the results to adolescents who are out of school. Besides that, as the DQIA-BR is calculated based on the 24hR, food consumption may not be reported properly, because it depends on memory, as well as smaller portions of foods known to be unhealthy or higher consumption of healthy foods.^30^ To minimize this bias, a second 24hR was performed in a subsample of the study to better estimate adolescents’ usual diet. In addition, other important variables, such as be on a diet or eating disorders, were not evaluated.

On the other hand, this study also has strengths. We can mention its sample size, its national representativeness, and the use of a diet quality index adapted to the Brazilian population and age group. Research also investigated the association of perception distortion and quality of diet in the different strata of nutritional status and raised important data on the food consumption of the adolescent population in Brazil in relation to their weight perception.

In conclusion, body weight perception seems to influence the consumption of healthy foods and the quality of diet, especially for adolescents who consider themselves overweight, but weight perception impacts the diet of adolescents differently according to their nutritional status. More nutritional education actions must be carried out among the young Brazilian population, especially regarding the nutritional quality of food, and awareness about the nutritional status and self-image as a form of comprehensive health promotion. Longitudinal studies are needed to better assess the impact of body weight perception in different dimensions of food throughout adolescence.
